# Supplementary material for: Limb-use by foraging marine turtles, an evolutionary perspective
Source: PeerJ. 2018 Mar 28;6:e4565. doi: 10.7717/peerj.4565 (PMC5878658; doi:10.7717/peerj.4565)
Supplement: Table S1 [file peerj-06-4565-s001.docx]

**Online Supplemental Material**

**Online Supplemental Material for Fujii. et al; “LIMB USE BY FORAGING MARINE TURTLES, AN EVOLUTIONARY PERSPECTIVE”**

In this supplemental material, we present more detailed information of the observed limb-use behaviors in marine tetrapods

**Table S1. List of marine tetrapods known to use limbs for feeding, prey type consumed and feeding habitat when behaviors were observed, limbs typically used for propulsion, and links to videos with examples of behaviors.**

| Family | Species | Limb Behavior | Limb Used in Feeding | Propulsion | Prey Type | Feeding Habitat | Videos of limb behavior*^Ŧ^* | Video Accessed Date |
| --- | --- | --- | --- | --- | --- | --- | --- | --- |
| Cheloniidae | *Eretmochelys imbricata* | Leveraging  Corralling  Holding | Foreflipper | Foreflippers | Porifera,  Cnidaria | Epipelagic  Sea grass  Coral reef | <https://youtu.be/TaJPBDRh7Wo>  <https://youtu.be/tocQlwgJITE> | Accessed 20 Nov 2017  Accessed 20 Nov 2017 |
|  | *Chelonia mydas* | Digging^[1]^  Leveraging  Swiping  Corralling | Foreflipper | Foreflippers | Sea grass,  Porifera,  Cnidaria,  Macroalgae | Coral reef | <https://youtu.be/7O7WNg9oNq8>  <https://youtu.be/L4x4bJI5mdo> | Accessed 20 Nov 2017  Accessed 20 Nov 2017 |
|  | *Caretta caretta* | Swiping^[2]^  Digging^[2]^ | Foreflipper | Foreflippers | Mollusca | Soft sediment | <https://youtu.be/XZUptZNkdSs> | Accessed 20 Nov 2017 |
| Trichechidae | *Trichechus manatus.* | Corralling ^[3]^  Digging^[3]^ | Foreflipper | Tail | Macroalgae | Soft sediment | <https://youtu.be/Cx5FrxGTOAQ?t=2m44s> | Accessed 20 Nov 2017 |
| Dugongidae | *Dugong dugon* | Corralling^[3]^ | Foreflipper | Tail | Sea grass | Sea grass |  |  |
| Delphinidae | *Orcinus orca* | Striking^[4]^  Tossing^10]^ | Tail | Tail | Pinniped  Fishes | Epipelagic | <https://youtu.be/G7WGIH35JBE> | Accessed 20 Nov 2017 |
|  | *Tursiops aduncus* | Kerplunking^[5]^ | Tail | Tail | Fishes | Soft sediment | <https://youtu.be/4BSC7tm1Efc?t=2m2s> | Accessed 20 Nov 2017 |
| Balaenopteridae | *Megaptera novaeangliae* | Lobtail^[6]^ | Tail | Tail | Fishes | Epipelagic |  |  |
| Mustelidae | *Enhydra lutris* | Holding^[7]^  Leveraging^[7]^  Digging^[7]^  Pounding^[7]^ | Forepaws | Hindflippers | Mollusca  Crustacea | Rocky Benthic  Kelp Forest  Soft sediment | <https://youtu.be/-HVNpndnf1M> | Accessed 20 Nov 2017 |
| Odobenidae | *Odobenus rosmarus* | Digging ^[8]^ | Foreflipper | Hindflippers | Mollusca | Soft sediment | <https://youtu.be/QiaOVYXbV7E> | Accessed 20 Nov 2017 |
| Otariidae | *Neophoca cinerea* | Holding^[9]^  Leveraging^[9]^ | Foreflipper | Foreflippers | Fishes | Epipelagic  Rocky Benthic | <http://onlinelibrary.wiley.com/doi/10.1111/mms.12384/suppinfo> | Accessed 20 Nov 2017 |
| Phocidae | *Phoca vitulina* | Holding ^[10]^  Digging ^[11]^ | Foreflipper | Hindflippers | Fishes | Epipelagic  Benthic | <https://youtu.be/Rq7O8DRZqZ4> | Accessed 20 Nov 2017 |
|  | *Halichoerus grypus* | Holding^[12]^ | Foreflipper | Hindflippers | Pinniped | Epipelagic | <https://vimeo.com/119776916> | Accessed 20 Nov 2017 |

***^Ŧ^***Videos were posted publicly on video platforms such as Youtube or Vimeo, found by searching for the species common name and the term “feeding” or “foraging”.

**SUPPLEMENT REFERENCES**

[1] Christianen, M.J., Herman, P.M., Bouma, T.J., Lamers, L.P., van Katwijk, M.M., van der Heide, T., Mumby, P.J., Silliman, B.R., Engelhard, S.L. & van de Kerk, M. 2014 Habitat collapse due to overgrazing threatens turtle conservation in marine protected areas. *Proc R Soc London B: Biol Sci* **281**, 20132890. (doi:10.1098/rspb.2013.2890).

[2] Schofield, G., Katselidis, K.A., Dimopoulos, P., Pantis, J.D. & Hays, G., C. 2006 Behaviour analysis of the loggerhead sea turtle Caretta caretta from direct in-water observation. *Endangered Species Research* **2**, 71-79.

[3] Marshall, C.D., Maeda, H., Iwata, M., Furuta, M., Asano, S., Rosas, F. & Reep, R. 2003 Orofacial morphology and feeding behaviour of the dugong, Amazonian, West African and Antillean manatees (Mammalia: Sirenia): functional morphology of the muscular-vibrissal complex. *Journal of Zoology* **259**, 245-260.

[4] Baird, R.W. & Dill, L.M. 1995 Occurrence and behaviour of transient killer whales: seasonal and pod-specific variability, foraging behaviour, and prey handling. *Can J Zool* **73**, 1300-1311.

[5] Gonzalez, A.F. & Lopez, A. 2000 'Kerplunking’: Surface fluke-splashes during shallow-water bottom foraging by bottlenose dolphins. *Mar. Mamm. Sci.* **16**, 646-653. (doi:10.1111/j.1748-7692.2000.tb00959.x).

[6] Weinrich, M.T., Schilling, M.R. & Belt, C.R. 1992 Evidence for acquisition of a novel feeding behaviour: lobtail feeding in humpback whales, Megaptera novaeangliae. *Anim. Behav.* **44**, 1059-1072. (doi:<https://doi.org/10.1016/S0003-3472(05)80318-5>).

[7] Riedman, M.L. & Estes, J.A. 1990 The sea otter (*Enhydra lutris*): behavior, ecology, and natural history. *US Fish and Wildlife Service Biology Report* **90**, 1-126.

[8] Levermann, N., Galatius, A., Ehlme, G., Rysgaard, S. & Born, E.W. 2003 Feeding behaviour of free-ranging walruses with notes on apparent dextrality of flipper use. *BMC Ecol* **3**, 9.

[9] Hocking, D.P., Ladds, M.A., Slip, D.J., Fitzgerald, E.M.G. & Evans, A.R. 2016 Chew, shake, and tear: Prey processing in Australian sea lions (Neophoca cinerea). *Mar Mamm Sci*, 1748-7692.

[10] Bonner, W.N. 1989 Seals and Man - a changing relationship. *Biol J Linn Soc* **38**, 53-60.

[11] Bowen, W.D., Tully, D., Boness, D.J., Bulheier, B.M. & Marshall, G.J. 2002 Prey-dependent foraging tactics and prey profitability in a marine mammal. *Mar Ecol Prog Ser* **244**, 235-245.

[12] van Neer, A., Jensen, L.F. & Siebert, U. 2015 Grey seal (Halichoerus grypus) predation on harbour seals (Phoca vitulina) on the island of Helgoland, Germany. *J. Sea Res.* **97**, 1-4. (doi:<https://doi.org/10.1016/j.seares.2014.11.006>).
