# Supplementary figures and images for: Limb-use by foraging marine turtles, an evolutionary perspective
Source: PeerJ. 2018 Mar 28;6:e4565. doi: 10.7717/peerj.4565 (PMC5878658; doi:10.7717/peerj.4565)

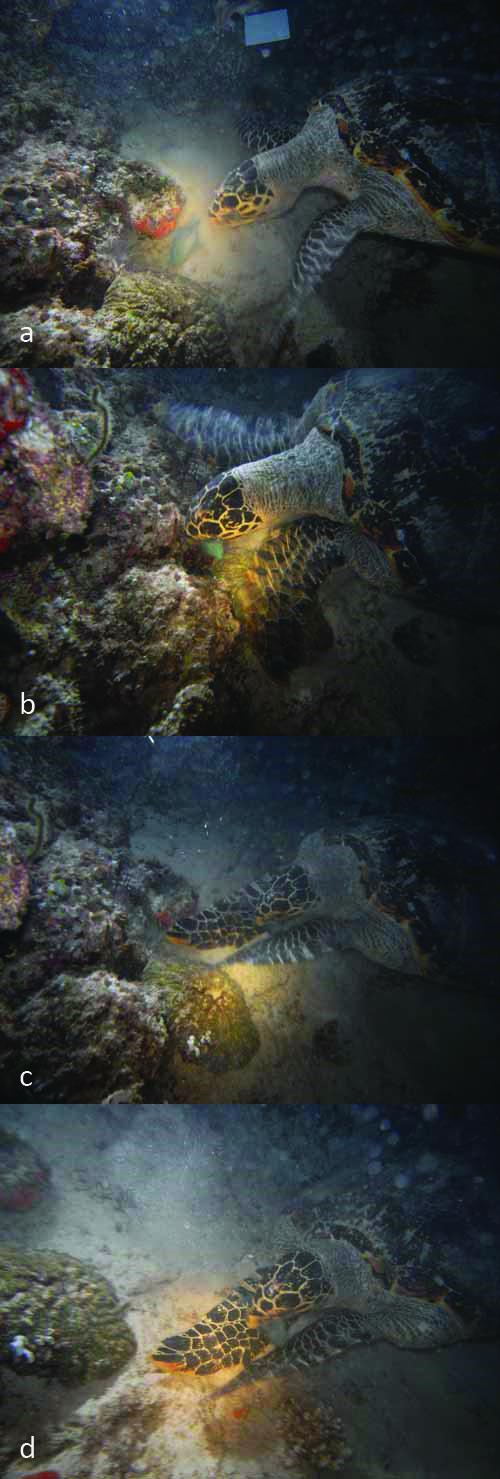

Supplement: Figure S1 — A series of frame grabs from a video recorded at night in Maldives shows a Hawksbill sea turtle using its foreflippers to capture and hold a red-toothed triggerfish (Odonus niger). Video shot by Simon Enderby, ©http://www.Scubazoocom and provided with permission from Dr. Nicolas Pilcher. [file peerj-06-4565-s002.jpg]
